# The effects of blueberry anthocyanins on histone acetylation in rat liver fibrosis

**DOI:** 10.18632/oncotarget.17842

**Published:** 2017-05-12

**Authors:** Wei Zhan, Xin Liao, Ru-Jia Xie, Tian Tian, Lei Yu, Xing Liu, Jing Liu, Po Li, Bing Han, Ting Yang, Bei Zhang, Li-Jun Cai, Rui Li, Qin Yang

**Affiliations:** ^1^ General Surgery of The Affiliated Hospital of Guizhou Medical University, Guiyang 550004, Guizhou Province, China; ^2^ Imaging Department of The Affiliated Hospital of Guizhou Medical University, Guiyang 550004, Guizhou Province, China; ^3^ Department of Physiology of The Guizhou Medical University, Guiyang 550004, Guizhou Province, China; ^4^ Department of Pathology of The Affiliated Hospital of Guizhou Medical University, Guiyang 550004, Guizhou Province, China; ^5^ Ultrasonic Center of The Affiliated Hospital of Guizhou Medical University, Guiyang 550004, Guizhou Province, China; ^6^ Department of Neurology of The Affiliated Hospital of Guizhou Medical University, Guiyang 550004, Guizhou Province, China; ^7^ Department of Recovery of the Guizhou People’s Hospital, Guiyang 550004, Guizhou Province, China

**Keywords:** blueberry, anthocyanin, hepatic stellate cell, liver fibrosis, histone acetylation

## Abstract

To determine the effects ofanthocyanins from blueberries on hepatic stellate cell (HSCs-T6) and on histone acetylation during liver fibrosis induced by CCl_4_ in rats. Fifty male SD rats weighing 180 ± 20g were randomly placed into a control group, a hepatic fibrosis group, a blueberry treatment group, a blueberry intervention group, and a natural recovery group. After the rats were sacrificed, the livers and the liver indexes were measured, and the pathological changes were observed by HE staining and Masson staining. The blood was analyzed for the four indexes of liver fibrosis and liver function; nucleoprotein from liver tissues and karyoplasm were isolated to determine the expression of acH3K9, acH3K14, and acH3K18 by Western blotting.

Compared with the lethal rate of the control group, the median lethal rate of HSCs-T6 cells treated with a the 50μmol/L concentration was 66.94% (*P* < 0.05). The protein expression on α-SMA, type I collagen, TIMP1 significantly decreased (*P* < 0.05) following treatment with 50 ug/ml of anthocyanin for 36 h; moreover, the expression of acH3K9, acH3K14 and acH3K18 modification were up-regulated (*P* < 0.05). Furthermore, compared with the liver in the model group, the liver in the intervention group showed the most obvious improvement (*P* < 0.01), and its karyoplasm had increased expression of acH3K9, acH3K14 and acH3K18 (*P*<0.01).

Regulating histone acetylation could improve liver function and liver fibrosis indexes in rats with hepatic fibrosis. The mechanism might be related to certain genes that promote apoptosis, so as to inhibit the effect of anti hepatic fibrosis.

## INTRODUCTION

It has been reported that histone acetylation may play a role in the development of hepatic fibrosis; however, the mechanism through which histone acetylation leads to the development and progression of hepatic fibrosis is unknown. It has been reported that blueberries can improve liver fibrosis through cell apoptosis; so what changes does histone acetylation undergo during the development of liver fibrosis? How does the level of histone acetylation change in the body and liver of blueberry-treated animals *in vitro* and *in vitro*? To answer these questions, we explored the effect of the anthocyanins in blueberries on the apoptosis of rat hepatic stellate cell lines (HSCs-T6), and the modification of acH3K9, acH3K14, and acH3K18 loci in this study; further, the effects of blueberries on liver function, hepatic fibrosis, and the histone acetylation of acH3K9, acH3K14, and acH3K18 were investigated in rats after liver fibrosis induced by carbon tetrachloride (CCl4)

Liver fibrosis is a process of repeated injury and repair of liver cells under the action of various pathogenic factors. In the liver, the extracellular matrix increases and the degradation decreases, which leads to a change in liver tissue structure and the appearance of liver fibrosis or cirrhosis [1-3]. In recent years, a considerable amount of research on liver fibrosis induced by HSC activation has been conducted, and more in-depth understanding of the process has been achieved. The role of epigenetic regulation in the process of liver fibrosis is, however, still in the exploratory stage. It has been shown that blueberries have the highest content of anthocyanins in fruits and vegetables [4]. Furthermore, it is known that anthocyanins are greatly significant to human health [5]. In this study, we observed the effect of the anthocyanins in blueberry on the cellular model *in vitro* and CCl_4_-induced liver fibrosis in the rat histone acetylation modification model, and provided a reference for the next experiment.

## RESULTS

### Extraction of anthocyanins from blueberries

**Figure 1:**
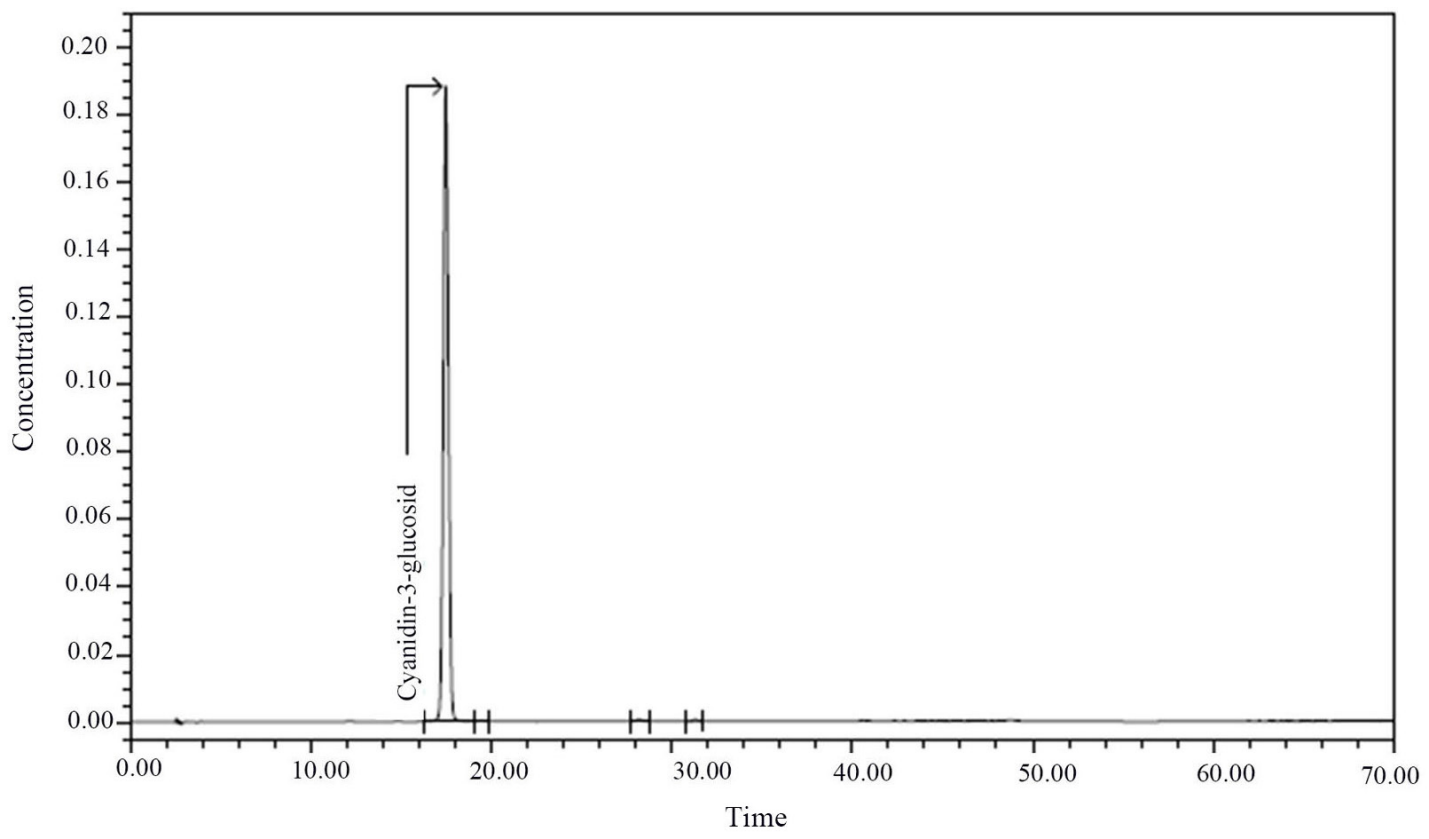
Chromatogram of Cyanidin-3-glucosid

Somerset [6] found that blueberries contained anthocyanins, including delphinidin, malvidin, peonidin, and petunidin. In Srivastava’s [7] study, it was found that blueberries contained anthocyanins mainly, such as cyanidin, delphinidin, malvidin and peonidin. There are at least 16 kinds of anthocyanin species in blueberries from Majiang County, Guizhou Province; however, we selected the cyanidin-3-glucosid for this study. After purification, its purity was 99%. The composition of anthocyanins from blueberries grown in Guizhou in the recent 3 years was analyzed and compared with the data from the mass spectrometry database. The results are shown in Table 1, Table 2, and Figure 1.

**Table 1:** Comparative analysis of the content of cyanidin-3-glucosid in Guizhou blueberry from 2011 to 2013

Year	The content of cyanidin-3-glucosid (mg/100g)
2011	308.94±806.56
2012	180.68±796.91^*^
2013	215.60±346.45^*^

*Vs* 2011, there are differences in 2012 and 2013(^*^P < 0.05); *Vs* 2012, there was no difference of anthocyanin content in 2013 (*P* > 0.05).

**Table 2:** Molar Absorptivity of Different Anthocyanins

Anthocyanin ^*a*^	Solvent system	λvis-max ^(nm)^	Molar absorptivity (ε)	Reference
*Cyanidin (Cyd)*				
Cyd	0.1% HCl in ethanol	510.5	24600	Schou, 1927
	0.1% HCl in ethanol	547	34700	**Ribereau-Gayon, 1959**
Cyd-3-ara	15:85 0.1 N HCl/ethanol	538	44400	Zapsalis and Francis, 1965
	15:85 0.1 N HCl/ethanol	535	44460	Fuleki and Francis, 1968a
Cyd-3,5-diglu	0.1 N HCl	520	30175	Niketic-Aleksic and Hrazdina, 1972
	Methanolic HCl	508.5	35000	Brouillard and El Hache Chahine, 1980
Cyd-3-gal	0.1% HCl in methanol	530	34300	Siegelman and Hendricks, 1958
	15:85 0.1 N HCl/ethanol	535	44900	Sakamura and Francis, 1961
	15:85 0.1 N HCl/ethanol	535	46200	Zapsalis and Francis, 1965
	15:85 0.1 N HCl/ethanol	535	46230	Fuleki and Francis, 1968a
	HCl in methanol	530	30200	Swain, 1965
Cyd-3-glu	Aqueous buffer, pH 1	510	26900	Jurd and Asen, 1966
	0.1 N HCl	520	25740	McClure, 1967
	1% HCl in methanol	530	34300	**Siegelman and Hendricks, 1958**
	10% ethanol, pH 1.5	512	18800	**Heredia et al., 1998**
Cyd-3-rut	Aqueous buffer, pH 0.9	510	7000	**Figueiredo et al., 1996**
	1% HCl	523	28840	Swain, 1965
Cyd-3-sam-5-glu	Aqueous buffer, pH 0.9	522	3600	Figueiredo et al., 1996
Cyd-3-sam-5-glu + sinapic + caffeic + malonic	Aqueous buffer, pH 0.9	538	21200	Figueiredo et al., 1996
Cyd-3-sam-5-glu + sinapic + ferulic	Aqueous buffer, pH 0.9	528	15100	Figueiredo et al., 1996
Cyd-3-sam-5-glu + sinapic + ferulic + malonic	Aqueous buffer, pH 0.9	538	20100	Figueiredo et al., 1996
Cyd-3-sam-5-glu + sinapic + p-coum + malonic	Aqueous buffer, pH 0.9	536	19000	Figueiredo et al., 1996
Cyd-3-soph-5-glu	Methanolic HCl	524	37150	Hrazdina et al., 1977
Cyd-3-soph-5-glu + malonic	Methanolic HCl	528	32360	Hrazdina et al., 1977
Cyd-3-soph-5-glu + sinapic	Methanolic HCl	528	37150	Hrazdina et al., 1977
Cyd-3-soph-5-glu + di-sinapic	Methanolic HCl	530	38020	Hrazdina et al., 1977
Cyd-3-soph-5-glu + ferulic	Methanolic HCl	528	32360	Hrazdina et al., 1977
Cyd-3-soph-5-glu + di-ferulic	Methanolic HCl	530	34670	Hrazdina et al., 1977
Cyd-3-soph-5-glu + p-coumaric	Methanolic HCl	526	38020	Hrazdina et al., 1977
Cyd-3-soph-5-glu + di-p-coumaric	Methanolic HCl	528	32360	Hrazdina et al., 1977
*Delphinidin (Dpd)*				
Dpd	0.1% HCl in ethanol	522.5	34700	Schou, 1927

### Effects of anthocyanins on proliferation of HSCs-T6 cells

**Figure 2:**
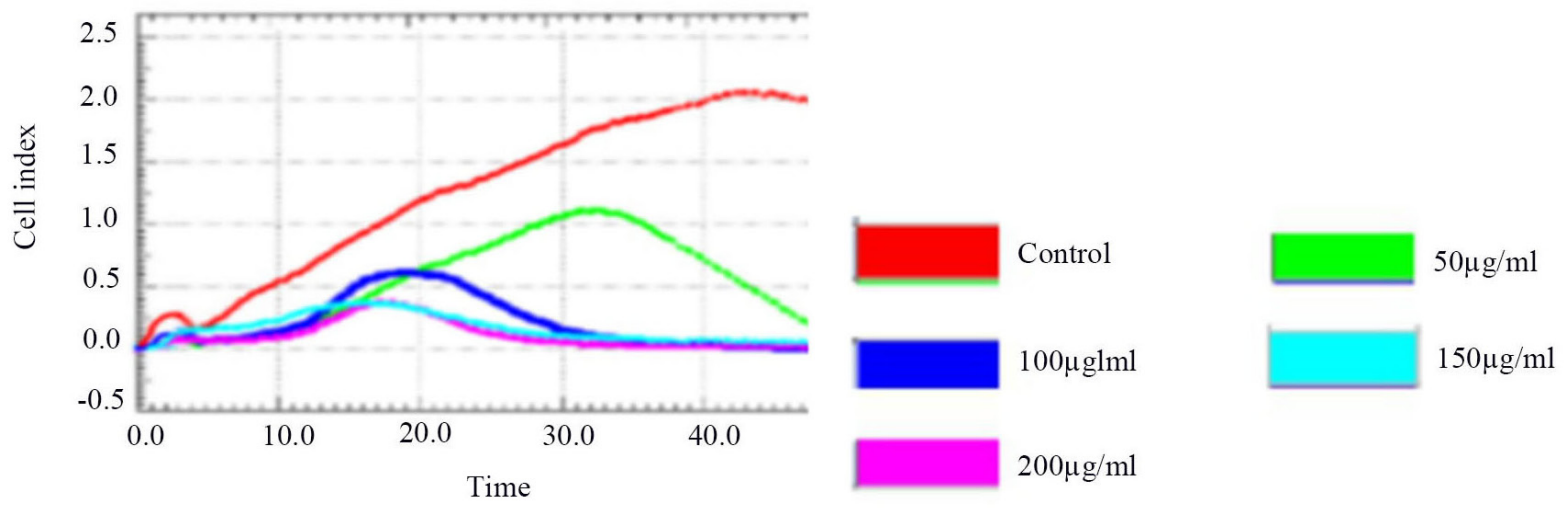
the RTCA of inhibition of HSCs-T6 proliferation by different concentration groups

Figure 2 and Table 3 show that 50 ug/ml, 100 ug/ml, 150 ug/ml, and 200 ug/ml of blueberry extract had a dose-dependent inhibitory effect on HSCs-T6 cells, and the difference was statistically significant (*P* <0.01). The 50 ug/ml group showed a more than 50% inhibition at 30 h of culture, while the rest of the concentration groups showed a more than 50% inhibition at 36 h; therefore, the 50 ug/ml blueberry anthocyanin concentration was selected for the experiment, and the experiment lasted 30 hours.

**Table 3:** The RTCA xCELLigence values of HSCs-T6 (%. Mean±SD. *n*=4)

	Co-culturing time	Control	Anthocyanin concentration			
			50ug/ml	100ug/ml	150ug/ml	200ug/ml
Cell index	36h	2.29±0.44	0.756±0.014^*^	0.526±0.007^*^	0.348±0.006^*^	0.217±0.007^*^
Inhibition rate	36h	1.00±0.00	0.669±0.006^*^	0.745±0.047^*^	0.848±0.003^*^	0.905±0.003^*^

^*^P<0.05 *νs* control group.

### Immunofluorescence results

**Figure 3:**
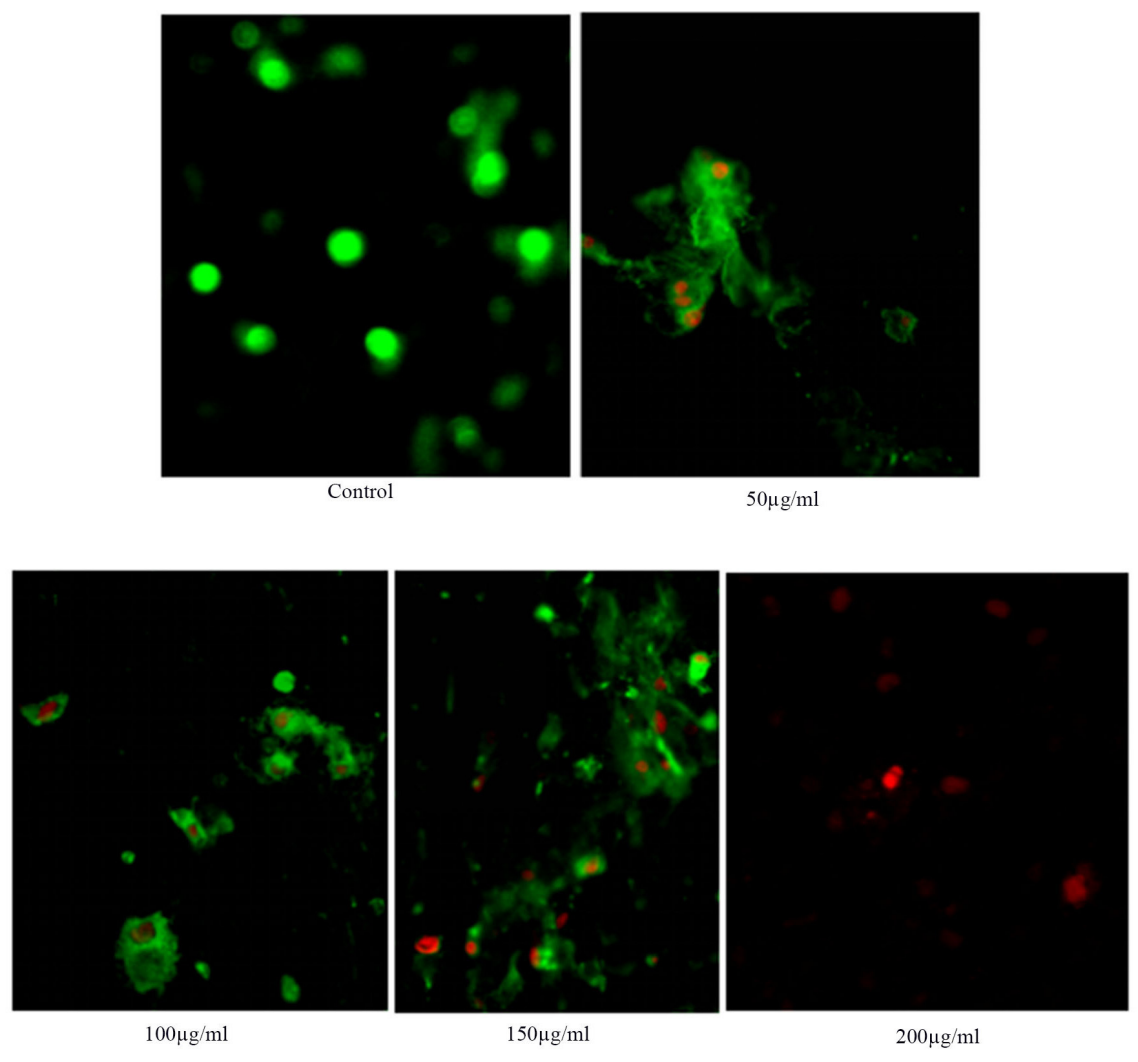
Detection of anthocyanin induced apoptosis in HSCs-T6 cells by Annexin V fluorimetric method

The apoptosis of HSCs-T6 cells treated with different concentrations of anthocyanins for 36 h at different concentrations was observed under a fluorescence microscope (Figure 3)

With the increase of blueberry anthocyanin concentration, HSCs-T6 apoptosis increased, and the nucleus red dye gradually increased. The results showed that blueberry anthocyanin had an inhibitory effect on HSCs-T6 cells; the difference was statistically significant (*P* < 0.01, and the effect was dose dependent.

### The role of apoptosis in HSCs-T6 cells

**Figure 4:**
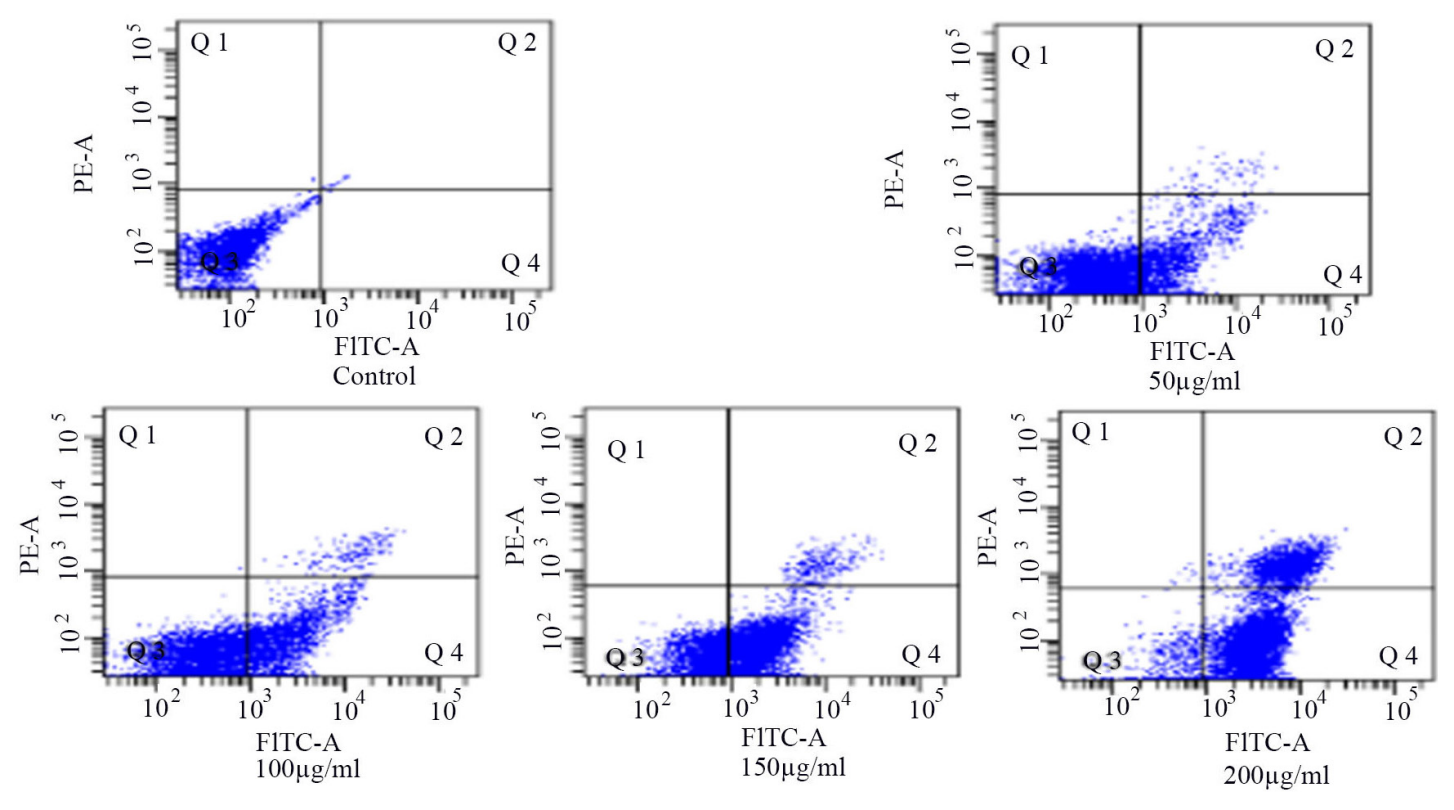
Detection of the apoptosis of HSCs-T6 cells treated with different concentrations of blueberry anthocyanins for 36h

Flow cytometry showed that the number of cells in the Q4 area increased gradually (Figure 4). The results showed that the apoptosis of HSCs-T6 increased with an increase in blueberry anthocyanin concentration, which indicated that blueberry anthocyanins had an inhibitory effect on HSCs-T6 cells. The difference was statistically significant (*P* <0.01), and the effect was dose-dependent.

### Expression of α-SMA, type I collagen, and TIMP1 protein in treated and control HSCs-T6 cells

The expression of α-SMA, TIMP1, and type I collagen in the control group and HSCs-T6 cells treated with 50 ug/ml of blueberry anthocyanin was detected by Western blot. The results showed (Figure 4) that expression of α-SMA, TIMP1, and type I in HSCs-T6 cells treated with 50 ug/ml of blueberry anthocyanin was lower than that in the control group. The difference was statistically significant (*P* <0.05).

### Histone acetylation modification expression of acH3K9,acH3K14,acH3K18 protein in treated and control HSCs-T6 cells

**Figure 5:**
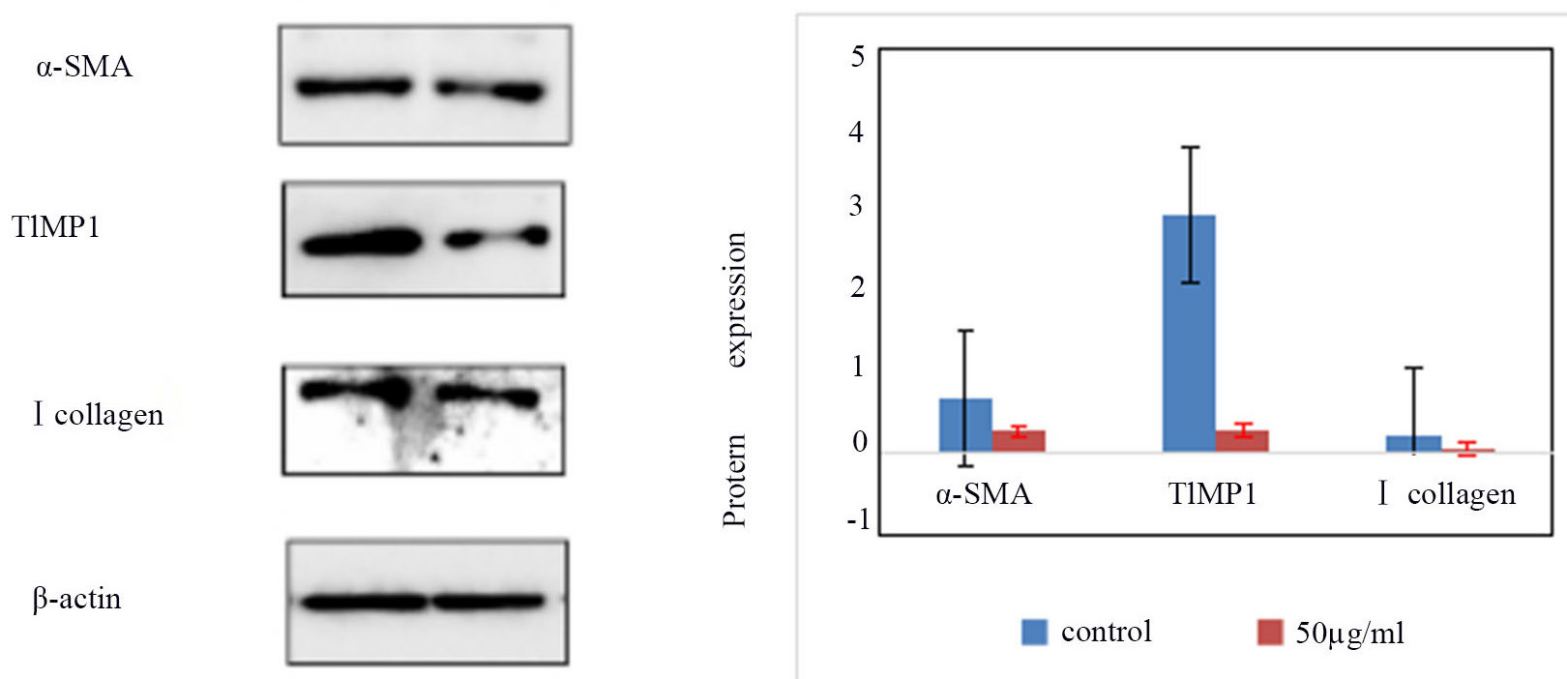
the expression of α-SMA, TIMP1, type I collagen in control group and HSCs-T6 cells treated with 50ug/ml of blueberry anthocyanin for 36h

**Figure 6:**
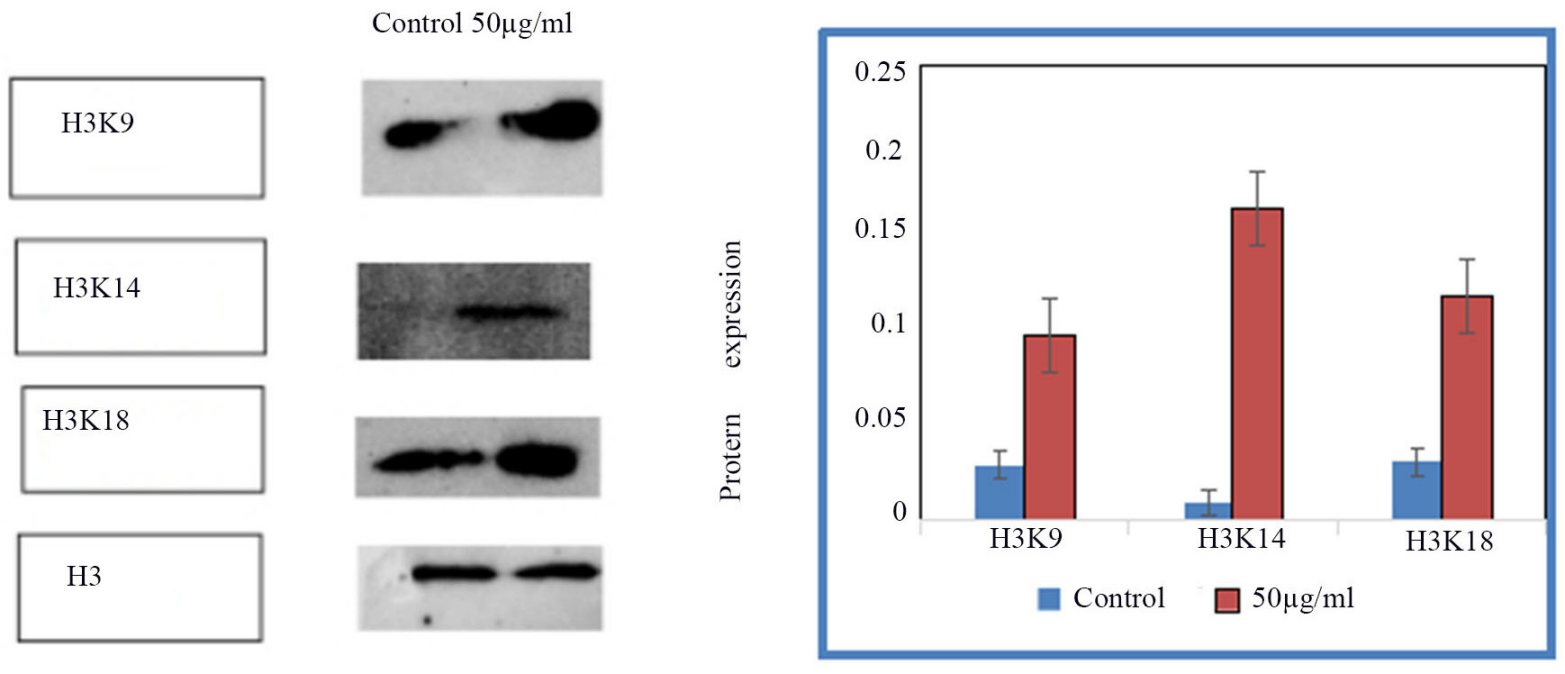
The expression of acH3K9, acH3K14, acH3K18 in control group and HSCs-T6 cells treated with 50ug/ml of blueberry anthocyanin for 36h

The expression of acH3K9, acH3K14, acH3K18 in the control group and HSCs-T6 cells treated with 50 ug/ml of blueberry anthocyanin was detected by Western blot. The results showed (Figures 5 and 6) that the expression of acH3K9, acH3K14, acH3K18 was higher in cells treated with 50 ug/ml of blueberry anthocyanin than in the control group. The difference was statistically significant (*P* < 0.01).

### Comparison of liver function in rats of each group

As shown in Table 4, compared with the normal group, the model group had significantly higher ALT values (*P* < 0.01); compared with the model group, the blueberry prevention and blueberry treatment groups had significantly lower ALT values (*P* < 0.01); compared with the natural recovery group, the blueberry prevention group had significantly lower ALT values (*P* < 0.01), and there was no significant difference between the blueberry treatment group and the natural recovery group (*P* > 0.05). The AST in the model group was significantly higher than that in the normal group (*P* < 0.01); compared with the model group, the blueberry prevention and blueberry treatment group had significantly lower AST values (*P* < 0.01); compared with the natural recovery group, the blueberry prevention group was significantly increased (*P* < 0.01), and there was no significant difference between the blueberry treatment group and the natural recovery group (*P* > 0.05).

**Table 4:** The levels of ALT and AST in rats of different groups ($\bar{x}$ ±s, n=8)

Group	ALT(U/L)	AST(U/L)
Normal	32.12±4.48	124.14±6.44
Liver fibrosis	1098.19±154.50^a^	673.21±72.38^a^
Blueberry treatment	102.85±22.07^b^	223.90±20.26^b^
Blueberry intervention	46.40±9.54^bc^	154.77±6.33^bc^
Natural recovery	121.79±5.94	270.37±27.54

^**a**^ vs Normal group;*P*<0.01. ^**b**^ vs Liver fibrosis group,*P*<0.05.

^**c**^vs Natural recovery group,*P*<0.05.

### Liver fibrosis four indicators results

As shown in Table 5, we determined levels of HA, CIV, LN, and PIIINP. Compared with the normal group, the model group had significantly increased levels of HA (*P* < 0.01); compared with the model group, the blueberry prevention and blueberry treatment group had significantly lower levels of HA (*P* < 0.01); compared with the natural recovery group, the blueberry prevention group had significantly lower levels of HA (*P* < 0.01), and there were no significant differences between the blueberry treatment group and the natural recovery group (*P* > 0.05).

**Table 5:** Four comparison of liver fibrosis in rats of each group ($\bar{x}$ ±s, n=8)

Group	HA(ng/ml)	LN(ng/ml)	PIIINP(ng/ml)	CIV(ng/ml)
Normal	104.19±2.87	0.23±0.01	0.00±0.00	0.20±0.03
Liver fibrosis	579.17±66.55^a^	0.20±0.00	0.00±0.00	1.27±0.06^a^
Blueberry treatment	206.69±15.44^b^	0.20±0.00	0.00±0.00	0.46±0.05^bc^
Blueberry intervention	135.70±7.47^bc^	0.20±0.00	0.00±0.00	0.34±0.06^bc^
Natural recovery	194.81±5.23	0.20±0.00	0.00±0.00	0.66±0.09

^**a**^ vs Normal group;*P*<0.01. ^**b**^ vs Liver fibrosis group,*P*<0.05.

^**c**^ vs Natural recovery group,*P*<0.05.

The levels of CIVin the model group were higher than those in the normal group (*P* < 0.01); compared with the model group, the blueberry prevention and blueberry treatment groups had significantly lower levels of CIV(*P* < 0.01); compared with the natural recovery group, the blueberry prevention group and the blueberry treatment group had significantly lower levels of CIV(*P* < 0.05). There were no significant difference in LN and PIIINP between the groups (*P* > 0.05).

### Pathological results

#### Comparison of HE and Masson staining of rat liver sections

**Figure 7:**
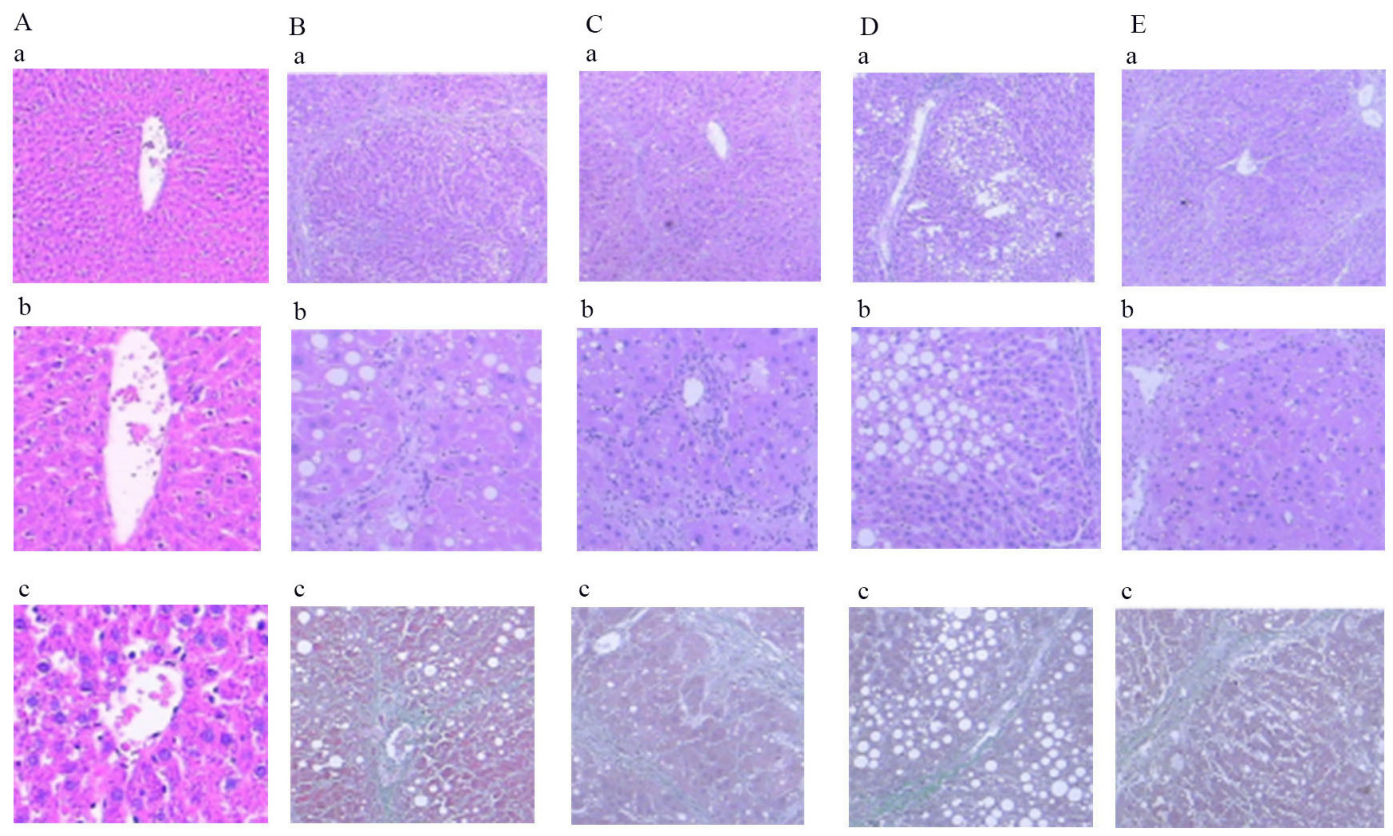
Comparison of HE and Masson staining in liver of rats in each group **(a-1)** Normal group: HE staining, ×40, normal hepatic cell morphology. **(b-1)** Modal group: HE staining, ×40, disruption of structure of hepatic lobules, false leaflet formation. **(c-1)** Treatment group: HE staining, ×40, the collagen fibers are linked to each other, and are wrapped around the liver. **(d-1)** Intervention group: HE staining, ×40, Collagen fibers extend and are not connected to each other. **(e-1)** Natural recovery group: HE staining, ×40, Collagen fibers extend and connect with each other. **(a-2)** Normal group: HE staining, ×100, normal hepatic cell morphology. **(b-2)** Modal group: HE staining, ×100, disruption of structure of hepatic lobules. **(c-2)** Treatment group: HE staining, ×100, complete destruction of hepatic leaflet structure. **(d-2)** Intervention group: HE staining, ×100, Collagen fibers to mild periportal extension. **(e-2)** Natural recovery group: HE staining, ×100, Collagen fibers extend significantly. **(a-3)** Normal group: Masson staining, ×200, normal hepatic cell morphology. **(b-3)** Modal group: Masson staining, ×100, fiber hyperplasia. **(c-3)** Treatment group: Masson staining, ×100, structural disorder of hepatic lobe. **(d-3)** Intervention group: Masson staining, ×100, Collagen fibers extend outward from the central veins. **(e-3)** Natural recovery group: Masson staining, ×100, Collagen fibers extend outward.

As shown in Figure 7, the liver fibrosis grade of the model group was significantly higher than that of the normal group (*P* < 0.01). Furthermore, the liver fibrosis grades of the blueberry prevention and blueberry treatment groups were significantly lower than that of the model group (*P* < 0.01). Compared with the natural recovery group, the blueberry prevention group had a significantly lower grade of liver fibrosis (*P* < 0.01); however, there was no significant difference in the level of hepatic fibrosis between the natural recovery group and the treatment group (*P* > 0.05).

### Expression of acH3K9, acH3K14, acH3K18

**Figure 8:**
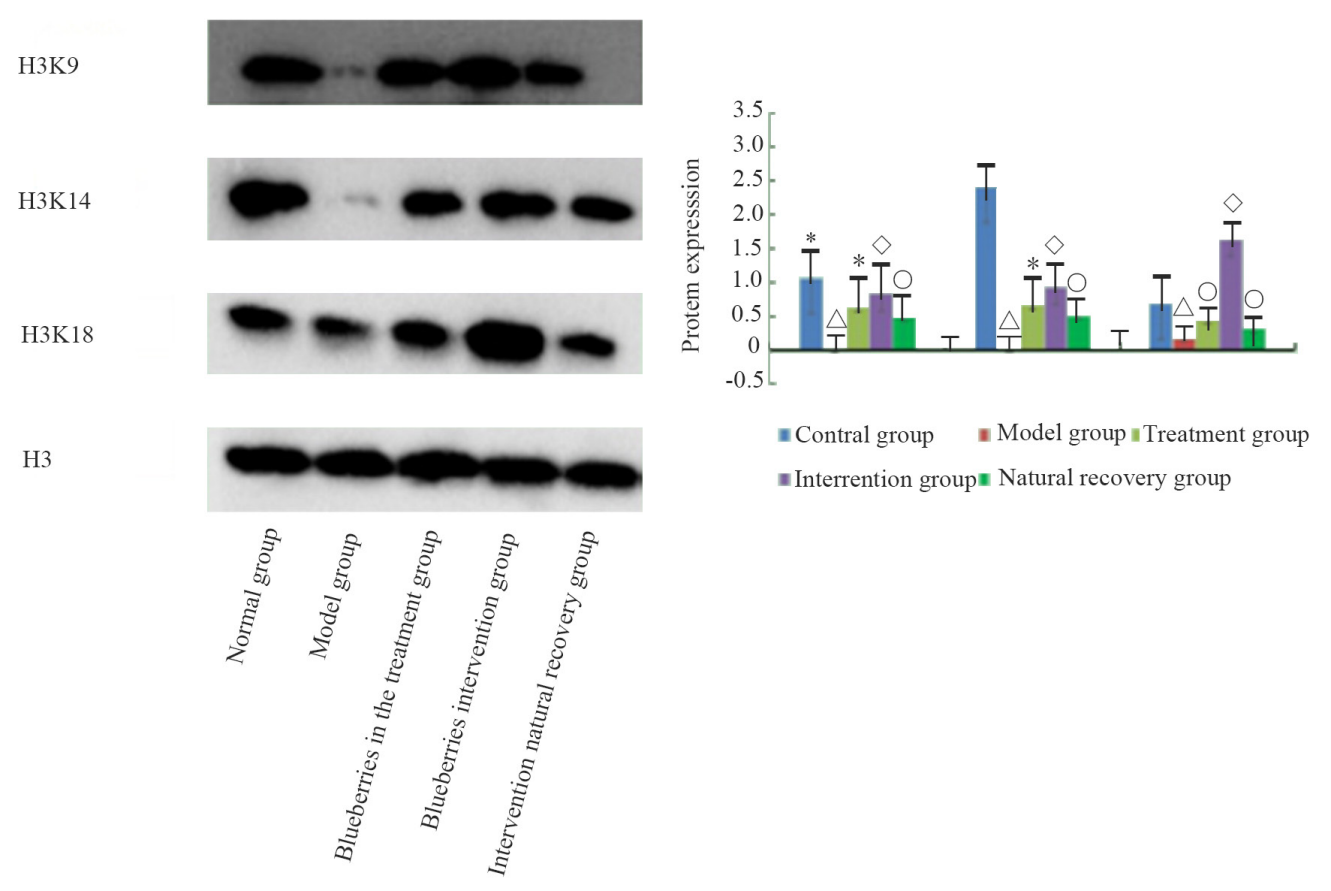
the protein modification of liver group of rats in each group

Comparison of the expression of acH3K9, acH3K14, acH3K18 modification in the liver of rats in each group is shown in Figure 8. The expressions of acH3K9, acH3K14, and acH3K18 proteins in the model group were lower than in the control group, and the levels of the three protein modifications were lower, and there were significant differences (*P* < 0.01). Compared with the model group, the blueberry treatment group, blueberry prevention group, and natural recovery group had higher levels of acH3K9, acH3K14, and acH3K18 protein (*P* < 0.01). Furthermore, the levels of acH3K9, acH3K14, and acH3K18 protein in the blueberry treatment group and the blueberry prevention group were significantly higher than those in the natural recovery group (*P* < 0.05). The results of the blueberry prevention group were more obvious.

## DISCUSSION

In recent years, the use of natural nutrients in food to prevent and treat liver fibrosis has attracted widespread attention. In this study, the medicinal properties of blueberries from Majiang County, Guizhou Province, were investigated. In particular, the effect of blueberries on histone acetylation in hepatic stellate cell lines *in vitro* was determined. Furthermore, the effect of blueberries on the acetylation of histone acetylation in liver fibrosis induced by carbon tetrachloride in rats was investigated.

Liver fibrosis is the abnormal proliferation of fibrous connective tissue in the liver under the persistent stimulation of chronic inflammation or injury, and is a common pathological characteristic of all chronic liver diseases. It is characterized by the increase in extracellular matrix (ECM) synthesis and secretion of type I and type III collagen in the liver, and the degradation is relatively insufficient, resulting in excessive ECM in the liver [2]. Some studies have suggested that the activation of hepatic stellate cells plays a key role in the development of hepatic fibrosis [8].

Hepatic stellate cell activation is the central link in the formation of hepatic fibrosis. Through intracellular signal transduction, continuous stimulation of hepatic stellate cell proliferation, the excessive synthesis and secretion of collagen, and other extracellular matrix deposition in the liver ultimately lead to the formation of liver fibrosis [9]. Studies had found that the activation of HSCs was difficult to reverse. However, by inducing apoptosis, it is possible to reduce the number of HSCs, reduce the synthesis of ECM, increase the degradation of ECM, and also induce the reversal of liver fibrosis [10].

CCl_4_ induced rat liver fibrosis model is a classic model of hepatic fibrosis. Hepatocyte injury and necrosis causes inflammatory cell aggregation and activation, releasing of a large number of cytokines. Furthermore, a considerable amount of ECM is deposited in the liver, which leads to the occurrence of liver fibrosis [1, 11].

Previous studies have found that there is endoplasmic reticulum stress in the course of the occurrence of liver fibrosis. At the same time, the expression of ERS marker protein-regulated protein glucose 78 is significantly increased. In addition, some studies had indicated that the rat spinal cord injury could inhibit the endoplasmic reticulum stress and promote cell apoptosis, improving the recovery of function [12].

Presently, several studies have focused on epigenetic inheritance, which can affect gene expression and genetic modification without changing the DNA sequence. Histone modifications include methylation, acetylation, phosphorylation, and ubiquitination [13, 14]. However, the degree and the state of these modifications determines whether a gene will be expressed [15]. Histone acetylation modifications are regulated by histone acetyltransferases and histone deacetylases, which depend on the dynamic equilibrium of HAT and HDAC [16]. Studies have indicated that high acetylation can neutralize the positive charge of lysine residues and reduce the contact between histone and DNA, which favors the binding of transcription factors and DNA [17, 18]. Histone acetylation modification occurs on site 9, 14, 18, and 23 of the H3 lysine residues and site 5, 8, 12, and 16 of the H4 arginine residues, which could be activated by gene silencing [19, 20]. H3K9 acetylation is an important mechanism that regulates gene transcription [21]. During histone acetylation, an acetyl group is transferred to a lysine rich residue group protein H3 or H4 under the action of histone acetyltransferase, neutralizing the positive charge and creating an environment that is conducive for the binding of transcription factors and DNA, and that promotes transcription [22-24].

Blueberries, also known as Vaccinium Ericaceae, contain antioxidant effects that can reduce the harmful effects of various diseases [25]. Srivastava [7] found that blueberry anthocyanins mainly contained cyanidin, delphinidin, malvidin, and peonidin. These substances could increase the apoptosis of cells and promote the recovery of the original function of cells. Related literature reported that blueberries could inhibit the activation of HSCs *in vitro* [26, 27], which this study reports as well. Barros [28] found that the anthocyanins in blueberries acted as antioxidants, protecting genes and preventing DNA from being damaged.

In the *in vitro* experiment, the anthocyanins of blueberry had a concentration- dependent effect on HSCs-T6 cell apoptosis, and apoptosis increased gradually with an increase in anthocyanin concentration. When the control group was compared with the 50 ug/ml HSC-T6 cells group, we observed that some specific indicators of α-SMA, collagen type I, and TIMP1 protein expression had reduced; however, the expression of acH3K9, acH3K14 and acH3K18 increased at the corresponding sites of histone acetylation. In the *in vivo* experiment, the model of liver fibrosis was induced by CCl_4_; ALT, AST and two important indicators of liver fiber had a more obvious effect in the blueberry intervention group compared with other groups, and it could be inferred from the results that long-term consumption of blueberries could improve liver fibrosis. The expression of acH3K9, acH3K14 and acH3K18 in histone acetylation could also be seen: the expression of acH3K9, acH3K14, and acH3K18 in the blueberry intervention group was significantly higher; there were obvious differences between this group and the treatment and the natural recovery groups. Although it is not definitive that some of the ingredients in blueberries could improve CCl_4_ induced liver fibrosis, we can speculate that long-term use of blueberries might increase the body’s antioxidant capacity and improve some indexes of histone acetylation in liver diseases. It can be inferred that the effective components of blueberry anthocyanins can restore the level of histone acetylation; however, whether this effect can be used as a potential inhibitor of histone acetylation is unknown, and it needs to be further explored and studied.

In addition, in the present study, we did not use primary activated hepatic stellate cells in rats, which may be a limitation of this study. In conclusion, the formation of liver fibrosis *in vivo* and *in vitro* showed that histone acetylation modification of acH3K9, acH3K14, and acH3K18 reduced in the presence of blueberry anthocyanins. *In vitro*, it was found that anthocyanins could promote the apoptosis of hepatic stellate cells. Further studies are needed to determine whether an inevitable link exists between the two observations.

## MATERIALS AND METHODS

### Materials

The blueberries were purchased from the Blueberry Planting Base, Majiang County, Guizhou Province. The blueberries were of the Lowbush blueberry Vaccinium Ericaceae strain, and their breed was blue circle. Following purchase, the blueberries were stored at -80°C in liquid nitrogen. HSCs-T6 cells were preserved in our laboratory; the male SD rats were 50, and their weights were 180 ± 20 g. Rats and feed were provided by the Animal Experimental Center of Guizhou Medical University.

### Main reagents

Gibco fetal bovine serum was purchased from Thermo Fisher Scientific; DMEM culture media was purchased from HyClone; Dnase I, and the rainbow marker were purchased from Solarbio; Cell lysates, protease inhibitors, BCA protein assay kit, Annexin V-FITC/PI cell apoptosis detection kit, Alexa Fluor488 labeled Goat anti mouse IgG (H+L), and DyLight549 labeled Goat anti rabbit IgG (H+L) were purchased from Shanghai Beyotime Biotechnology Co. Ltd.; Rabbit Anti-alpha smooth muscle actin polyclonal antibody was purchased from Beijing Boosen Biological Technology Co., Ltd.; TIMP1 monoclonal antibody and Anti-Collagen Type Rabbit I polyclonal antibody were purchased from Bioworld; acH3, acH3K9, acH3K14, and acH3K18 antibodies were purchased from Abcam. Real time cellular analysis was provided by Hangzhou Essen biological Co. Ltd.

### Blueberry anthocyanin extraction method

We selected 2011–2013 year blue circle variety of blueberries grown in Majiang County, Guizhou Province, and weighed and pan milled them. The extraction solvent was 1% hydrochloric acid (HCl) in methanol. The milled blueberries were dissolved in the extraction solvent for 2 h. The ratio of milled blueberries to extraction solvent was 1:4. The mixture was centrifuged at 4000 r/min for 15 min, and then filtered with a 0.25 um filter. The hydrochloric acid and methanol residues were removed by rotary evaporation at 40°C. The crude extract was purified by macroporous resin AB-8, and the purified anthocyanins were identified by high performance liquid chromatography (HPLC-MS) (6). The composition of anthocyanins in the blueberries was analyzed and compared with the data from the mass spectrometry database. Nanjing Plant Research Institute of Chinese Academy of Sciences had used the same method to extract more than 16 kinds of anthocyanins from blueberries; this experiment selected cornflower -3-O- glucoside, and its purity was 99%, as shown in Figure 1.

### Cell proliferation

The cells were cultured at 37°C in culture media containing 10% fetal bovine serum, DMEM, and penicillin and streptomycin in 5% CO_2_, and the media was regularly changed. Log growth phase cells were used for the experiment.

Log growth phase of HSCs-T6 cells were taken, and the 50 ul DMEM was added to the E-Plate. The baseline reading was obtained to ensure that the Index Cell of each well was less than 0.063. The following different concentrations of blueberry anthocyanins were added to a 100 ul suspension of HSCs-T6 cell in the detection well: 50, 100, 150, 200 ug/ml. The number of cells per cell was 5000 cells/ 100 ul. At the same time, the control group was set up with cells that were not treated with anthocyanins. Detection was achieved using a Station RTCA. The ICELLigenic system was used to detect cell adherence, growth, and proliferation of cells every other 15 min and after 72 h. Cell index (index cell) was used to detect the cell impedance parameters, and the experiment was repeated 3 times. The IC50 of HSCs-T6 cells treated with blueberry anthocyanins was determined. Inhibition rate (%) = (Normal group - experimental group) / Normal group × 100%.

### In situ fluorescence of anthocyanin on HSCs-T6 cells apoptosis

The logarithmic growth phases of HSCs-T6 cells were taken into the 3 cm no bags of plastic dishes spread 1×10^5^ cells. The cells were cultured in the incubator at 5% CO_2_. When the cell wall was about 80%, the culture media was aspirated, and the cell layer was washed 3 times with PBS. Then, whole culture media containing 10% fetal bovine serum was added to the dishes that contained different concentrations of anthocyanin (50, 100, 150, 200 ug/ml). The control group was treated with whole culture medium not containing the drug. After culture, the morphology of HSC-sT6 cells was observed under the light microscope. Then, Annexin V-FITC binding buffer, Annexin V-FITC, PI were added to the dishes in volumes of 15 ul/ dish, 5 ul/ dish, and 10 ul/ dish. The incubation occurred in the dark at room temperature for 20 min. The cell morphology was observed with an inverted fluorescence microscope.

### Apoptosis rate of HSCs-T6 cells

The 5 × 10^5^ HSCs-T6 cells were cultured in 10 cm plastic culture dishes without package. When the cell wall was more than 80%, the experimental group was treated with different concentrations of anthocyanins in complete culture medium, and the control group treated with the culture medium without the drug. The cells were cultured in the incubator at 5% CO_2_; then the old culture solution was aspirated and the cell layer was washed 3 times with PBS. The cells were digested with trypsin without EDTA, and the cell pellet was suspended again with PBS. After the cell count, 5–10 million cells were added to 15 ul of V-FITC binding buffer, 5 ul of Annexin V-FITC, and 10 ul of PI. After being gently re-suspended, the cells were placed in the darkroom, where they were incubated at room temperature for 15 min. They were then analyzed by flow cytometry.

### Expression of α-SMA, type I collagen and TIMP1 protein in HSCs-T6 cells

The cells were collected, added to the protein lysis solution, and placed an ice for 10 min. Then, they were centrifuged for 12000 r/min at 4°C for 20 min. The precipitate was discarded, and the supernatant was collected. The protein was quantified by the BCA protein quantitative kit, according to the provided manual. After protein quantification, the 40 ug samples were exposed to polyacrylamide gel electrophoresis, and then transferred to a cellulose nitrate membrane. They were then immersed in 5% skim milk powder, and 1:500 diluted Rabbit anti α-SMA antibody, a 1:400 dilution of Rabbit anti collagen antibody, and a 1:500 dilution of Rabbit anti TIMP1 antibody were added. The membranes were shaken at 4°C overnight. On the second day, after TBST washing, the membranes were incubated with the secondary antibody anti labeled with horseradish peroxidase (1:4000) at room temperature for 90 min, and washed 3 times with the TBST. ECL light emission imaging was used as the internal reference, with beta-actin as the internal reference. The Image lab.lnk analysis software was used to analyze the gray value of each band.

### Expression of acH3K9, acH3K14, acH3K18 protein in HSCs-T6 cells

Proteins were extracted from the cells of each group. After protein quantification, the 40 ug samples were exposed to polyacrylamide gel electrophoresis, and then transferred to a cellulose nitrate membrane. They were immersed in 5% skim milk powder and a 1:200 dilution of Rabbit anti acH3K9 antibody, a 1:400 dilution of Rabbit anti acH3K14 antibody, and a 1:400 dilution of Rabbit anti acH3K18 antibody were added. The membranes were shaken at 4°C overnight. On the second day, after TBST washing, the membranes were incubated with secondary antibody anti labeled with horseradish peroxidase (1:4000) at room temperature for 90 min, and washed 3 times with the TBST. ECL light emission imaging was used as the internal reference, with acH3 as the internal reference. The Image lab.lnk analysis software was used to analyze the gray value of each band.

### Rat model making method

SD male rats and feeds were provided by the Animal Experimental Center of Guizhou Medical University. The rats subsisted on a free diet and were given sufficient feed and water. In this experiment, 50 male rats were randomly divided into 5 groups, with 10 rats in each group. The liver fibrosis model group was injected with 40% carbon tetrachloride vegetable oil solution 0.3 ml/100 g once every three days, which established the model of liver fibrosis (the injections were halted at the end of 8 weeks, n = 10). The rats in the normal control group were injected subcutaneously with the same amount of vegetable oil (the rats were sacrificed at the end of 8 weeks). For the blueberry treatment group, 8 weeks after the liver fibrosis model was started, 5 ml of blueberry juice was administered to the stomach of the rats 2 times a day for 8 weeks (a total of 16 weeks; n = 10). For the blueberry intervention group, 4 weeks before the model was halted, the mice were given 5 ml of blueberry juice to fill their stomach, 2 times a day, for 8 weeks(a total of 12 weeks. n = 9). For the natural recovery group, after 8 weeks of liver fibrosis modeling, the rates were given a normal diet for 8 weeks (n = 9).

### Specimen collection

Rats were sacrificed under strict conditions. After hydration with chlorine aldehyde, the rats were weighed. The rats were fixed on an anatomical plate, and blood vessels from the left iliac blood vessels along the left groin region were removed. The liver was excised and weighed.

### Pathological and related index detection method

The left lobe of the liver was immersed in 4% neutral formaldehyde; HE and Masson staining were completed in the Affiliated Hospital of Guizhou Medical University. Liver function (ALT and AST) and liver fiber (HA, LN, PIIINP, CIV) were detected by the Affiliated Hospital of Guizhou Medical University and the Liver Disease laboratory. Residual liver was stored in a -80°C fridge.

### Extraction and separation of plasma protein and nuclear protein from karyoplasm

A microbalance was used to accurately weigh 60 mg of rat liver tissue. The Blood (Bioworld) Karyoplasmic Separation Kit was used to extract plasma protein and nuclear protein. Quantization of nuclear proteins was achieved using the BAC Protein Quantization Kit.

### Western blot

Forty micrograms of nuclear protein and plasma protein were separately collected by SDS, and then transferred to a PVDF film after electrophoresis. According to the conventional method, the first antibody and the second antibody were then added to the reaction. After incubation with ECL light reagent for 1–2 min, the blot was exposure imaged using BIO-RAD gel imaging instrument. The experiment was repeated 3 times for each group, and the expression of related proteins was detected.

### Statistical analysis

The data was analyzed with SPSS 20 statistical software. Data was shown as mean ± SD. One factor analysis of variance (ANOVA) was used among the multiple groups, and the least significant difference method (LSD method) was used between the two groups. The differences were statistically significant when *P* < 0.05.
